# Scdrake: a reproducible and scalable pipeline for scRNA-seq data analysis

**DOI:** 10.1093/bioadv/vbad089

**Published:** 2023-07-06

**Authors:** Jan Kubovčiak, Michal Kolář, Jiří Novotný

**Affiliations:** Laboratory of Genomics and Bioinformatics, Institute of Molecular Genetics of the Czech Academy of Sciences, Vídeňská 1083, 142 20 Prague 4, Czech Republic; Laboratory of Genomics and Bioinformatics, Institute of Molecular Genetics of the Czech Academy of Sciences, Vídeňská 1083, 142 20 Prague 4, Czech Republic; Department of Informatics and Chemistry, Faculty of Chemical Technology, University of Chemistry and Technology in Prague, Technická 5, 166 28 Prague 6, Czech Republic; Laboratory of Genomics and Bioinformatics, Institute of Molecular Genetics of the Czech Academy of Sciences, Vídeňská 1083, 142 20 Prague 4, Czech Republic; Department of Informatics and Chemistry, Faculty of Chemical Technology, University of Chemistry and Technology in Prague, Technická 5, 166 28 Prague 6, Czech Republic

## Abstract

**Motivation:**

While the workflow for primary analysis of single-cell RNA-seq (scRNA-seq) data is well established, the secondary analysis of the feature-barcode matrix is usually done by custom scripts. There is no fully automated pipeline in the R statistical environment, which would follow the current best programming practices and requirements for reproducibility.

**Results:**

We have developed scdrake, a fully automated workflow for secondary analysis of scRNA-seq data, which is fully implemented in the R language and built within the drake framework. The pipeline includes quality control, cell and gene filtering, normalization, detection of highly variable genes, dimensionality reduction, clustering, cell type annotation, detection of marker genes, differential expression analysis and integration of multiple samples. The pipeline is reproducible and scalable, has an efficient execution, provides easy extendability and access to intermediate results and outputs rich HTML reports. Scdrake is distributed as a Docker image, which provides a straightforward setup and enhances reproducibility.

**Availability and implementation:**

The source code and documentation are available under the MIT license at https://github.com/bioinfocz/scdrake and https://bioinfocz.github.io/scdrake, respectively.

**Supplementary information:**

Supplementary data are available at *Bioinformatics Advances* online.

## 1 Introduction

Single-cell RNA-seq (scRNA-seq) is a technology that is able to capture transcriptional profiles of thousands of individual cells (Islam *et al.*, 2011; Mereu *et al.*, 2020). Analysis of scRNA-seq data still remains challenging, mainly because of the technology itself, and partly due to the plethora of analysis tools developed and the lack of gold standards (Luecken and Theis, 2019). While there are currently well-established tools and pipelines for the initial quantification part of the analysis [e.g. 10× Genomics Cell Ranger (Zheng *et al.*, 2017)], the secondary analysis of the acquired feature-barcode matrix usually requires custom scripts and combinations of different software packages.

Several pipelines offering the secondary analysis of scRNA-seq data have been already published (see Supplementary Table S1). However, some of them lack current best analysis practices, are missing important steps of the analysis or offer only a graphical user interface without the full automation. Moreover, the majority is not implemented in a pipeline toolkit, and thus, is neither reproducible nor scalable, and does not provide access to intermediate results.

Here, we introduce scdrake, an automated pipeline for the secondary analysis of scRNA-seq data, which is fully implemented in the R statistical environment. Scdrake provides all important steps of the secondary analysis of a feature-barcode matrix: quality control, cell and gene filtering, normalization, detection of highly variable genes, dimensionality reduction, clustering, cell type annotation, detection of marker genes, differential expression analysis and integration of multiple samples. For those steps, we followed the best practices described in Amezquita *et al.* (2020) using the current state-of-the-art packages from the Bioconductor project (Huber *et al.*, 2015), namely, scran (Lun *et al.*, 2016), scater (McCarthy *et al.*, 2017), scDblFinder (Germain *et al.*, 2022), SC3 (Kiselev *et al.*, 2017), SingleR (Aran *et al.*, 2019), batchelor (Haghverdi *et al.*, 2018) and Seurat (Hao *et al.*, 2021). The pipeline is extensively configurable and provides rich graphical outputs and reports in HTML format. Internally, scdrake is an R package built upon drake (Landau, 2018), a Make-like pipeline toolkit for R. Thus, the pipeline is highly reproducible and scalable, has an efficient execution, provides easy access to intermediate results and is arbitrarily extendable. We strived to achieve maximum practicability for bioinformaticians while providing ample and meaningful outputs for biologists. At the same time, bioinformaticians can quickly react to biologists’ needs by changing the parameters of the pipeline, which then efficiently skips already finished parts. This dialogue between the biologist and the bioinformatician is indispensable during scRNA-seq data analysis. Scdrake ensures that this communication is performed in an effective and reproducible manner.

## 2 Results

### 2.1 Pipeline overview

**Figure 1.**
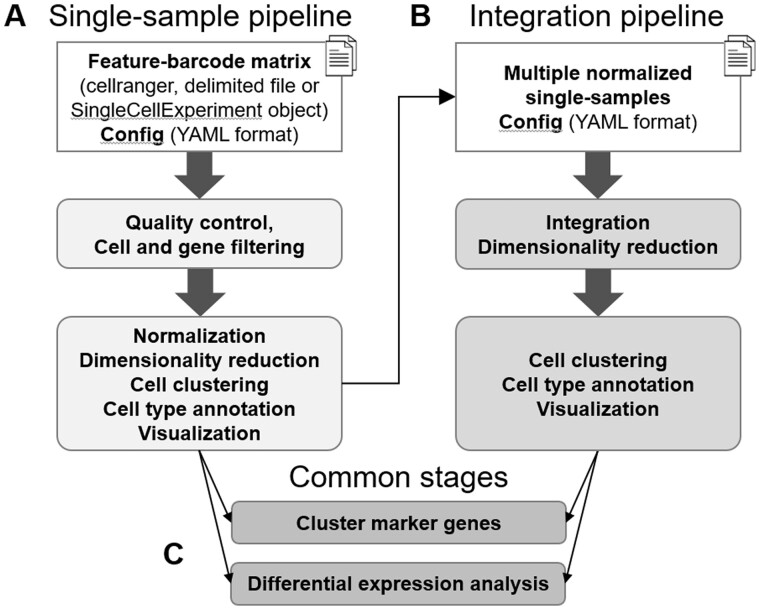
Overview of the scdrake workflow. The workflow consists of two pipelines: the first one performs analysis of an individual sample (A) while the second one integrates multiple independent samples (B). Stages for detection of cluster marker genes and differential expression analysis are shared by both pipelines (C). More details are provided in Supplementary Figs S1 and S2

The scdrake workflow (Fig. 1 and Supplementary Figs S1 and S2) consists of two pipelines, which are further divided into stages. Each stage finishes with a standalone HTML report (e.g. clustering or identification of marker genes). Users can use predefined standard tools and parameters or modify them for their particular needs.

**Figure 2.**
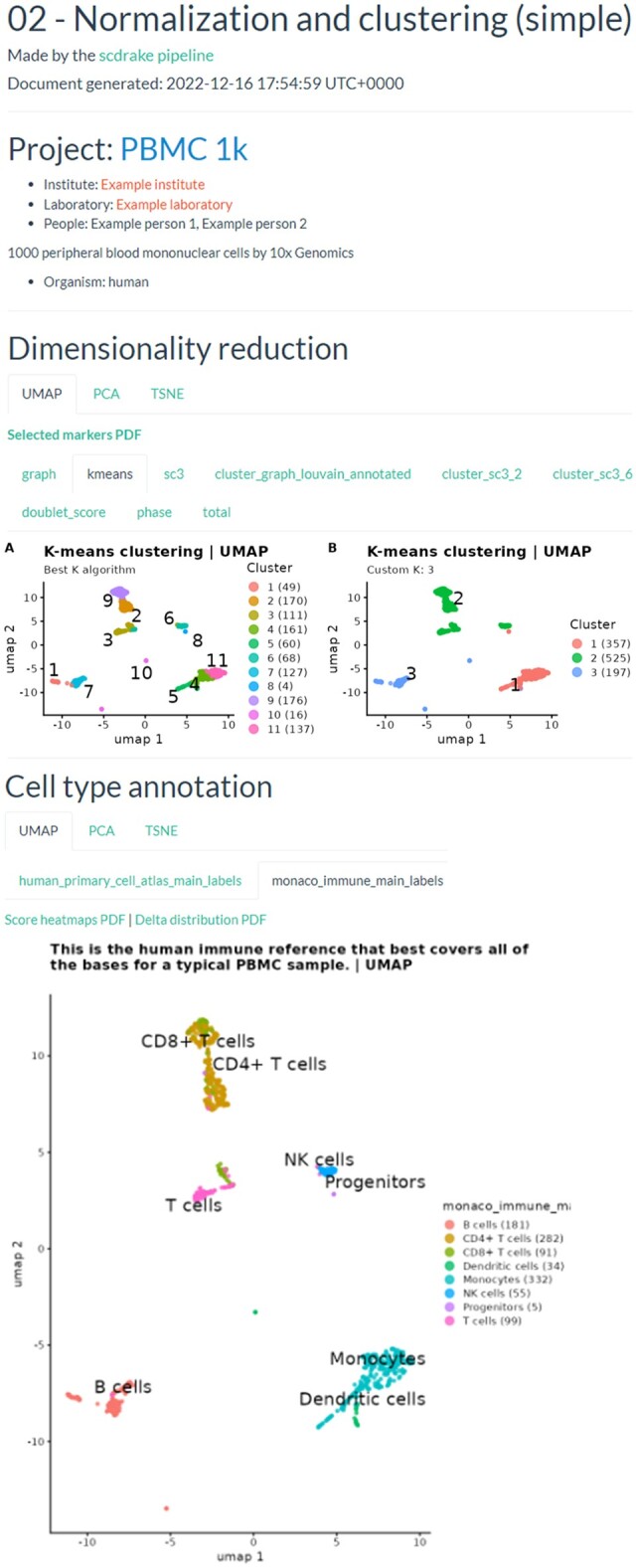
An example of the normalization and clustering stage HTML output from the single-sample pipeline. Users can view clustering results and any cell covariate [e.g. cell cycle phase or total number of unique molecular identifiers (UMIs) per cell] requested in the configuration in three different dimensionality reduction coordinates [uniform manifold approximation and projection (UMAP), t-distributed stochastic neighbour embedding (t-SNE), principal component analysis (PCA)]. These can be also used to display labels from the automatic cell annotation via SingleR. In the actual report, images link to full-size PDFs

The first pipeline (Fig. 1A) performs analysis of an individual sample. It starts with a feature-barcode matrix (in various formats) and contains two stages: quality control and filtering, followed by normalization, dimensionality reduction, clustering and cell type annotation. The second pipeline (Fig. 1B) integrates multiple independent samples, which were preprocessed by the single-sample pipeline previously. Its first stage performs sample integration *per se*, while the second step is similar to the second stage of the single-sample pipeline, except it works with the integrated data. Stages for detection of cluster marker genes and differential expression analysis are shared by both pipelines (Fig. 1C). More detailed information about the structure of scdrake’s pipelines can be found at https://bioinfocz.github.io/scdrake/articles/pipeline_overview.html.

We have tested the pipeline using the dataset of peripheral blood mononuclear cells (PBMCs 1K; 10× Genomics, CITE) and provided full outputs on the scdrake documentation page (https://onco.img.cas.cz/novotnyj/scdrake/). In Figure 2, we present an example output of the normalization and clustering stage of the single-sample pipeline.

### 2.2 Implementation details

Scdrake applies a project-based analysis approach; therefore, a new analysis starts with the initiation of a project directory, which includes all necessary configuration files, RMarkdown templates for stage reports and initial scripts for drake. Configuration files are stored in language-agnostic YAML ain’t markup language (YAML) format.

As the scdrake pipelines are in whole implemented in the R language using the drake toolkit, the pipeline definitions are in the form of R objects where each pipeline step (called *target*) is a piece of R code. Thus, scdrake can be used both from within R or through a simple command-line interface which wraps its most important R functions. When the pipeline is executed, drake constructs a dependency tree of targets and efficiently runs and saves to cache only targets whose code or upstream dependencies have changed since the last execution.

During runtime, drake recognizes which targets are currently independent of other targets and can be run in parallel (implicit parallelism). These abilities greatly enhance the execution time. In addition, users can load each target from a cache and implement custom pipelines that reuse the existing targets in scdrake (modular design). The majority of targets use data structures from Bioconductor, e.g. SingleCellExperiment, making it easy to use them within other Bioconductor packages or export them to other widely used formats.

As scdrake depends on many other packages, it is, for the sake of reproducibility, necessary to track their exact versions. For this purpose, scdrake utilizes the renv package (Ushey, 2022) that can restore exact package versions using a lock file, which is tracked by git. Moreover, to facilitate the installation of scdrake, we provide a Docker image in which all dependencies are installed. The Docker image is the most reproducible and straightforward way of how to use scdrake, and we recommend it to all users. We provide a separate vignette on the Docker image installation and usage (also within Singularity). Additional modes of installation are available for those, who cannot use the Docker image.

## 3 Conclusions

Scdrake provides a completely R-based, fully automated pipeline for the secondary analysis of scRNA-seq data, covering its most important parts. The pipeline is implemented using the drake toolkit that provides significant benefits in terms of reproducibility, speed, efficiency, access to intermediate results and modularity. We believe scdrake will become a popular choice for bioinformaticians and, in turn, its outputs interesting and useful for biologists.

Scdrake is available at https://github.com/bioinfocz/scdrake and its extensive documentation is at https://bioinfocz.github.io/scdrake. We are committed to further extending scdrake capabilities, mainly by including more analysis modules, such as gene set enrichment analysis, trajectory inference or support for spatial transcriptomic data. The project is open to the community and we gladly welcome all contributions.

## Supplementary Material

vbad089_Supplementary_Data
