# Supplementary material for: Scdrake: a reproducible and scalable pipeline for scRNA-seq data analysis
Source: Bioinform Adv. 2023 Jul 6;3(1):vbad089. doi: 10.1093/bioadv/vbad089 (PMC10351969; doi:10.1093/bioadv/vbad089)

Supplementary Figure 2: Low-level diagram of scdrake pipelines

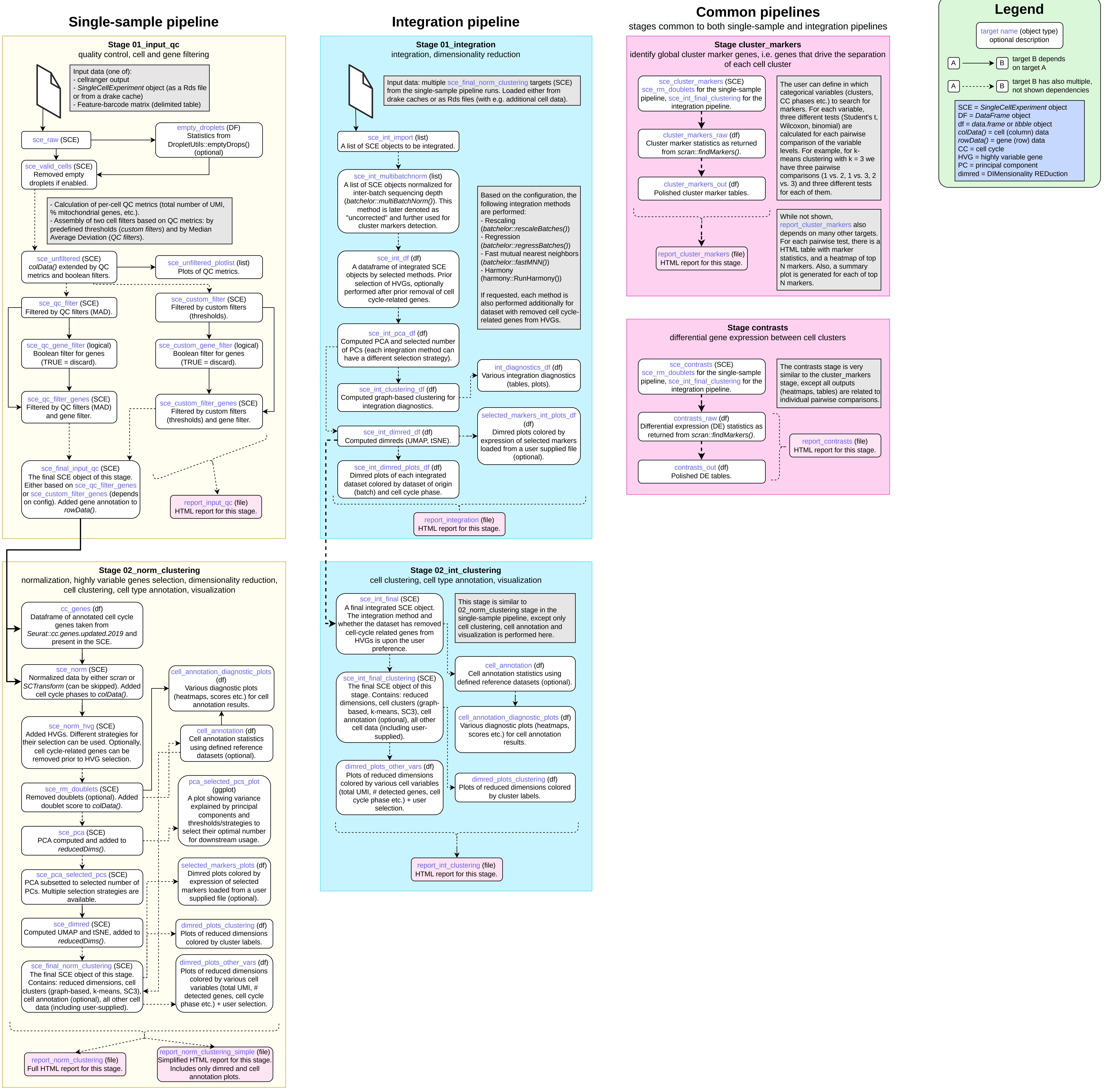

Supplement: vbad089_Supplementary_Data [file vbad089_supplementary_data.zip › suppl_fig_2.pdf]
