# Supplementary material for: Scdrake: a reproducible and scalable pipeline for scRNA-seq data analysis
Source: Bioinform Adv. 2023 Jul 6;3(1):vbad089. doi: 10.1093/bioadv/vbad089 (PMC10351969; doi:10.1093/bioadv/vbad089)
Supplement: vbad089_Supplementary_Data [file vbad089_supplementary_data.zip › suppl_fig_1.pdf]

Supplementary Figure 1: High-level overview of scdrake pipelines and methods

Note: the pipeline implementation does not consist of linear workflow as depicted here - some parts are branched and can be automatically computed concurrently.

Abbreviations

SCE = *SingleCellExperiment* object
CC = cell cycle
HVG = highly variable gene
PC = principal component
dimred = DIMensionality REDuction

Single-sample pipeline

Stage 01_input_qc
quality control, cell and gene filtering

Input data (one of):
- cellranger output
- *SingleCellExperiment* object
- Feature-barcode matrix (delimited table)

1. Read raw data.
2. Removal of empty droplets (optional). `DropletUtils::emptyDrops()`
3. Calculation of per-cell QC metrics, e.g. number of UMI and detected genes, or percentage of expressed mitochondrial genes. `scater::perCellQCMetrics()`
4. Calculation of boolean, QC metrics-based cell filters by two methods:
   - Dataset sensitive (called *QC*) filters: based on Median Average Deviation.
   - Custom filters: based on user defined thresholds.
5. Cell filtering separately for each filter (optional).
6. Gene filtering (optional) based on minimum number of UMI per cell and minimum ratio of cells expressing a gene.
7. Plots of QC metrics with visualization of differences between dataset-sensitive and custom filtering.
8. Final selection of the filtered dataset, added gene annotation from organism database (e.g. *org.Hs.eg.db).*
9. Visualization and HTML report.

Stage 02_norm_clustering
normalization, highly variable genes selection, dimensionality reduction, cell clustering, cell type

1. Calculation of cell cycle score and assignment of cells to phases (G1, G2M, S). `Seurat::cc.genes.updated.2019` `Seurat::CellCycleScoring()`
2. Normalization by either:
   - Deconvolution: `scran::computeSumFactors()` `scuttle::logNormCounts()`
   - Regularized negative binomial regression: `Seurat::SCTransform()`
3. Selection of HVGs by a selected metric (gene variance, coefficient of variation) and picking strategy (top N, thresholding on significance (FDR) or metric value). Optionally prior to HVG selection, detect and remove CC-related genes by their variance explained for CC phase. If normalization is performed by SCTransform, it automatically ranks genes and picks defined top N HVGs. `scran::modelGeneVar()` `scran::modelGeneCV2`
4. Detection and removal of cell doublets (optional). `scDblFinder::computeDoubletDensity()`
5. Calculation of PCA. `scater::runPCA()`
6. Selection of top N PCs by variance explained for downstream usage (dimensionality reduction (UMAP, tSNE), clustering). Three different strategies for selection of PCs are used and visualized:
   - Using the elbow point.
   - Using the technical noise.
   - Using the fixed number of PCs.
7. Cell clustering using:
   - Graph-based clustering (mutual nearest neighbors) using Louvain and Walktrap algorithms. `scran::buildSNNGraph()` `igraph::cluster_louvain()` `igraph::cluster_walktrap()` `igraph::cluster_leiden()`
   - K-means clustering using both custom numbers of K and best K selection.
   - Single-Cell Consensus Clustering (SC3) with custom numbers of clusters. `SC3::sc3()`
8. Cell type annotation (optional) using *celldex* package-provided or custom reference datasets. `SingleR::SingleR()`
9. Dimensionality reduction by UMAP and tSNE. `scater::runUMAP()` `scater::runTSNE()`
10. Visualization and HTML report.

Integration pipeline

Stage 01_integration
integration, dimensionality reduction

Input data: multiple sce_final_norm_clustering targets (SCE) from the single-sample pipeline runs. Loaded either from drake caches or as Rds files (with e.g. additional cell data).

1. Read datasets.
2. Subset to common column (cell variables) and row data (genes), and metadata.
3. Normalization of each sample for inter-batch sequencing depth (later called as "uncorrected" integration method). `batchelor::multiBatchNorm()`
4. Combine and select HVGs by either (and optionally remove CC-related genes prior to HVG selection):
   - A HVG metric (gene variance or CV2), then apply top HVGs selection as in the single-sample pipeline. `scran::combineVar()` `scran::combineCV2()`
   - Set operations (without duplicates): intersection, union, or take them all.
5. Perform integration by selected methods (and each separately for datasets with removed CC-related genes from HVGs, if enabled):
   - Rescaling: scale counts so that the average count within each batch is the same for each gene. `batchelor::rescaleBatches()`
   - Regression: fit a linear model to each gene regress out uninteresting factors of variation, returning a matrix of residuals. `batchelor::regressBatches()`
   - Fast mutual nearest neighbors. `batchelor::fastMNN()`
   - Harmony `harmony::RunHarmony()`
6. For each integrated dataset with or without removed CC-related genes from HVGs (if enabled), perform the following:
   - Calculate PCA and select number of PCs (same as in the 02_norm_clustering stage).
   - Calculate reduced dimensions (UMAP, tSNE) (same as in the 02_norm_clustering stage).
   - Mutual nearest neighbors (MNN) clustering to assess integration results.
   - Calculate integration diagnostics (tables, plots, Rand indices). `bluster::pairwiseRand()`
7. Visualization and HTML report.

Stage 02_int_clustering
cell clustering, cell type annotation, visualization

1. Select the final integrated dataset. This is upon the user preference, i.e. the integration method and whether CC-related genes were removed from HVGs (if enabled previously).
2. Perform the same procedures as in the 02_norm_clustering stage:
   - Cell clustering.
   - Cell type annotation.
3. Visualization and HTML report.

Common pipelines
stages common to both single-sample and integration pipelines

Stage cluster_markers
identify global cluster marker genes, i.e. genes that drive the separation of each cell cluster

1. Calculate statistical tests. The user can define in which categorical variables (clusters, CC phases etc.) to search for markers. For each variable, three different tests (Student's t, Wilcoxon, binomial) are calculated for each pairwise comparison of the variable levels. For example, for k-means clustering with k = 3 we have three pairwise comparisons (1 vs. 2, 1 vs. 3, 2 vs. 3) and three different tests for each of them. `scran::findMarkers()`
2. For each pairwise test, generate a HTML table with marker statistics, and a heatmap of top N markers and a summary plot for each of them.
3. Visualization and HTML report.

Stage contrasts
differential gene expression between cell clusters

1. Calculate statistical tests. The contrasts stage is very similar to the cluster_markers stage, except all outputs (heatmaps, tables) are related to individual pairwise comparisons. `scran::findMarkers()`
2. For each contrast and statistical test, generate a HTML table with marker statistics, and a heatmap of top N markers and a summary plot for each of them.
3. Visualization and HTML report.
